# Patient-Derived Explants as a Precision Medicine Patient-Proximal Testing Platform Informing Cancer Management

**DOI:** 10.3389/fonc.2021.767697

**Published:** 2021-12-20

**Authors:** Abby R. Templeton, Penny L. Jeffery, Patrick B. Thomas, Mahasha P. J. Perera, Gary Ng, Alivia R. Calabrese, Clarissa Nicholls, Nathan J. Mackenzie, Jack Wood, Laura J. Bray, Ian Vela, Erik W. Thompson, Elizabeth D. Williams

**Affiliations:** ^1^ School of Biomedical Sciences at the Translational Research Institute (TRI), Queensland University of Technology (QUT), Brisbane, QLD, Australia; ^2^ Centre for Personalised Analysis of Cancers (CPAC), Brisbane, QLD, Australia; ^3^ Queensland Bladder Cancer Initiative (QBCI), Brisbane, QLD, Australia; ^4^ Australian Prostate Cancer Research Centre - Queensland (APCRC-Q), Brisbane, QLD, Australia; ^5^ Department of Urology, Princess Alexandra Hospital (PAH), Brisbane, QLD, Australia; ^6^ Department of Medical Oncology, Princess Alexandra Hospital (PAH), Brisbane, QLD, Australia; ^7^ School of Mechanical, Medical and Process Engineering, Queensland University of Technology (QUT), Brisbane, QLD, Australia; ^8^ Australian Research Council (ARC) Training Centre for Cell and Tissue Engineering, Queensland University of Technology (QUT), Brisbane, QLD, Australia

**Keywords:** precision medicine, patient-derived explants, whole exome sequencing, *ex vivo*, cancer

## Abstract

Precision medicine approaches that inform clinical management of individuals with cancer are progressively advancing. Patient-derived explants (PDEs) provide a patient-proximal *ex vivo* platform that can be used to assess sensitivity to standard of care (SOC) therapies and novel agents. PDEs have several advantages as a patient-proximal model compared to current preclinical models, as they maintain the phenotype and microenvironment of the individual tumor. However, the longevity of PDEs is not compatible with the timeframe required to incorporate candidate therapeutic options identified by whole exome sequencing (WES) of the patient’s tumor. This review investigates how PDE longevity varies across tumor streams and how this is influenced by tissue preparation. Improving longevity of PDEs will enable individualized therapeutics testing, and thus contribute to improving outcomes for people with cancer.

## Introduction

Precision cancer medicine incorporates genetic sequencing results in the process of identifying optimal treatments for patients (1). Standard of care (SOC) therapies are typically prescribed based on anatomical origin and histological subtypes, however, this is not always the most effective treatment (1). Identifying genomic aberrations assists in understanding pathways driving tumor progression, which are often directly linked to the hallmarks of cancer (1–3). Understanding the functional significance of driver mutations in the context of a specific tumor is critical when moving towards clinical implementation of precision medicine techniques (1). Thus, there is a significant need for a patient-proximal *ex vivo* model reflecting the complex inter- and intra-tumor heterogeneity of the microenvironment (4, 5). An *ex vivo* model accurately reflecting patient responses to treatment will assist in identifying the druggable ‘Achilles’ heel’ of individual cancers. PDEs have been a focus of research for several types of solid tumor. However, the use of tissue explants is not limited to cancer research, with this culture system being used for research of several non-malignant human diseases including those of the liver (6) and inflammatory bowel disease (7).

## Patient-Derived Explants and Cellular Viability

PDEs are physiologically representative of the patient’s disease (8, 9). They represent a significant advance in patient-derived models when compared to two-dimensional (2D) cell lines and complement other three-dimensional (3D) models such as tumoroids/organoids [extensively reviewed in (10, 11)]. Major benefits of PDEs over other patient-proximal models include response prediction to clinical therapies, where cultures can be generated rapidly post-surgery and used to gauge a patient’s response to various anti-cancer therapies, and a more accurate reflection of the intrinsic tumor microenvironment (TME) (12). A faithful recapitulation of inter- and intra-tumor heterogeneity within the TME is key for precision medicine and is often not replicated in other organotypic models (13). Longevity of the various components which comprise the TME (including immune cells, stromal cells, and tumor cells), *ex vitro*, is an important consideration when utilizing these models to investigate adjuvant and neoadjuvant treatments (14).

An intact TME allows for metabolically active tumor, stromal and immune cells, preserving their native cellular interactions (14). Consequently, PDEs may more closely reflect responses to SOC and molecularly determined therapeutics compared to other patient-derived models such as cell lines, tumoroids/organoids, or patient-derived xenografts (PDX). The importance of monitoring the presence and longevity of immune cells within PDEs harboring pro-immunogenic or immunosuppressive environments has become increasingly recognized with findings that tumor infiltrating immune cells (TIICs) are faithfully retained in PDEs (8, 15). In pancreatic PDEs, CD45^+^ TILs, including cytotoxic T lymphocytes (CTLs; CD3^+^, CD8^+^), regulatory T cells (Tregs; CD3^+^, FOXP3^+^) and macrophages (CD68^+^, CD163^+^, HLA-DR^+^) have been shown to be viable for 6 to 9 days in culture (8, 15, 16). Notably, long-term culture of ovarian cancer PDEs retained macrophages, Tregs, CTLs, and B cells (CD19^+^, CD20^+^) up to 21-days in agitation culture (17). Most studies, however, do not systematically describe the immune compartments and lack details regarding the longevity of other resident adaptive and innate/innate-like immune subsets, including natural killer cells, γδ T cells and myeloid-derived suppressor cells. The ability to quantify specific immune cell subtypes in PDEs is key to interpreting functional relevance and will ensure these platforms redefine preclinical testing of immunotherapies.

Important disadvantages of PDEs include the relative short-term viability of the cultures evident in some tumor types (**Table 1**) such as endometrial PDEs which were viable for 1 day (23). The lack of a functional vascular system has been suggested as the reason for this, as it impacts oxygen diffusion and waste removal (34–36). Indeed, Lee, You (26) observed the development of hypoxic cores in head and neck squamous cell carcinoma (HNSCC) PDEs, likely due to a lack of oxygen and nutrient diffusion through the explant. Despite the use of PDE culture systems dating back to the 1980s (37), these models have not been widely adopted into clinical practice due to this short-term viability. However, several groups, working across multiple tumor streams, have been investigating new approaches to improve and extend explant viability (summarized in **Table 1**) to accommodate whole exome sequencing (WES) turnaround times and thus enable functional testing of genomically identified drug targets.

**Table 1:** PDE culture conditions for various tumor types.

Tumor Type	Culture Method	Media Base	Serum	Antibiotic (+/-)	Other Additives	Culture period***	Reference
Brain	PDEs placed on Millipore membrane inserts	MEM (Gibco)	Horse (Gibco)	+	Hank’s Balanced Salt Solution (ThermoFisher Scientific), L-glutamine (Gibco), glucose (Mediatech Inc.)	13 days	(18)
	PDEs placed on Millipore membrane inserts	MEM (Gibco)	–	+	Hank’s Balanced Salt Solution (Gibco), N-hydroxysuccinimide (Gibco), L-glutamine (Braun), glucose (Braun)	16 days	(19)
Breast	PDEs placed on gelatin sponges	RPMI1640^#^	Bovine^#^	+	Hydrocortisone^#^, insulin^#^	6 days	(20)
	PDEs free-floating	DMEM (Invitrogen)	Bovine^#^	–	GlutaMAX™ (Seralab)	7 days	(21)
	PDEs free-floating under orbital rotation	DMEM/Ham’s F12^#^	Bovine^#^	+	Hydrocortisone, insulin, Transferrin, 3, 3’, 5 Triidothyronine, epidermal growth factor (EGF), cholera toxin, adenine^#^	7 days	(22)
Endometrial	PDEs placed on Millipore inserts	DMEM/Phenol red free Ham’s F-12 (Sigma-Aldrich)	–	–	L-glutamine (Gibco/Invitrogen)	1 day	(23)
Head and Neck Squamous Cell Carcinoma	PDEs free-floating	DMEM (PAN Biotech-GmbH)	Bovine (PAN Biotech-GmbH)	+	Glutamine^#^	2 days	(24)
	PDEs placed on membrane inserts	RPMI1640** (Bio & Sell)	Bovine (Invitrogen)	+	Sodium bicarbonate, HEPES, cystine^#^	6 days	(25)
	PDEs placed on cell sheets and transferred to transwell membrane inserts	DMEM/Ham’s F12 (ThermoFisher Scientific)	Bovine (Sigma-Aldrich)	+	Insulin (Sigma-Aldrich), triiodothyronine (Sigma-Aldrich), adenine (Sigma-Aldrich), hydrocortisone (Sigma-Aldrich), recombinant EGF (ThermoFisher)	10 days	(26)
Lung	PDEs placed on Millipore insert discs	DMEM^#^	-/Bovine*^#^	+	Glucose^#^	3 days	(27)
	PDEs placed on Millipore filters	RPMI1640 (Gibco)	Bovine (Gibco)	+	Glutamine (Gibco)	3 days	(28)
	PDEs placed on Millipore insert	RPMI1640 (ThermoFisher Scientific)	Bovine (ThermoFisher Scientific)	+	L-glutamine (ThermoFisher Scientific)	12 days	(29)
Ovarian	PDEs placed on gelatin sponges	RPMI1640^#^	Bovine^#^	+	N/A	5 days	(30)
	PDEs free-floating under orbital rotation	DMEM (Gibco)	Bovine (Gibco)	+	N/A	30 days	(17)
Pancreatic	PDEs placed on Millipore inserts	CMRL1066	Human (Sigma-Aldrich)	+	HEPES (Gibco), sodium pyruvate (Gibco), zinc sulfate (Sigma-Aldrich), insulin-transferring-sodium selenite solution (Gibco), diphenyldiselenide (Sigma-Aldrich)	4 days	(31)
	PDEs placed on Millipore inserts precoated in collagen gel matrix	RPMI1640 (Gibco)	Bovine (Invitrogen)	+	EGF (Gibco), hydrocortisone (Sigma-Aldrich), insulin (Roche)	5 days	(15)
	PDEs placed on Millipore inserts coated in collagen matrix	RPMI1640 (Gibco)	Bovine (Gibco)	+	GlutaMAX™ (Gibco), sodium bicarbonate (Sigma-Aldrich), HEPES (Sigma-Aldrich), L-cystine (Sigma-Aldrich)	9 days	(8)
	PDEs placed on gelatin sponges	DMEM (Sigma-Aldrich)	Bovine (Sigma-Aldrich)	+	GlutaMAX™, hydrocortisone, insulin (Sigma-Alrich)	12 days	(16)
Prostate	PDEs placed on gelatin sponges	RPMI1640^#^	Bovine^#^	+	Hydrocortisone^#^, insulin^#^	6 days	(20)
	PDEs placed on nitrocellulose filters (32)	DMEM (Gibco)	Bovine (Gibco)	+	D-glucose, pyruvate (Gibco)	4 days	(32)
Uterine Leiomyomas	PDEs placed on Algine scaffold discs	DMEM (Biowest)	Bovine (Lonza)	+	L-Glutamine (Sigma-Aldrich)	7 days	(33)

*Compared different concentrations of serum.

**Phenol red and riboflavin free media.

***Maximum viable culture period reported

CMRL, Connaught Medical Research Laboratories; DMEM, Dulbecco’s Modified Eagle Medium; HEPES, 4-(2-hydroxyethyl)-1-piperazineethanesulfonic acid; -, not present; ^#^Source of material is not indicated in publication.

PDEs can be generated by fragmentation or slicing of fresh tumor tissue. Slice thickness affects the length of time a PDE is viable in culture. A range of different slice thicknesses have been used (200 µm to 3 mm), although in most cases these do not appear to have been empirically determined. Naipal, Verkaik (22) investigated different tissue slice thickness in breast cancer but did not identify an optimal thickness suitable to all tumor types. Additionally, Parajuli and Doppler (36) assessed the effect of slice thickness on longevity using MMTV-neu mouse tumors, determining that a thickness of 160 µm maintained tissue architecture and culture viability most effectively for this tumor type.

## Culturing Conditions

There is a lack of a consistent explant culturing methodology in the PDE literature. **Table 1** summarizes the variety of culture techniques used across tumor types. Investigating universal principles and best practice approaches that could be applied across tumor types, including the incorporation of tumor-specific components would facilitate the integration of this precision medicine pipeline into clinical practice.

### Tissue Preparation

A variety of tissue preparation strategies have been used to generate PDEs. The most common slicing methods are manual dissection with a scalpel and mechanized sectioning using either the vibratome (a vibrating blade microtome) or the McIlwain Tissue Chopper. Manual slicing is used to dissect excess fat or generate PDEs within a specific size range (30), however, it is becoming less common due to the lack of uniformity in explant thickness (16, 22). Two studies have directly compared the most common mechanized slicing technologies, the vibratome and Tissue Chopper, and found the Tissue Chopper more accurately and reproducibly produced slices of a specified thickness (19, 25). Furthermore, Holliday, Moss (21) were unable to slice breast tissue using a microtome due to the fatty nature of the tissue. By contrast, breast explants were successfully sliced using the Tissue Chopper resulting in improved tissue integrity when compared to a vibratome (22). Thus, where available, the McIlwain Tissue Chopper is currently the preferred approach for accurate slicing of tissues of varying densities.

### Supplements

Supplements incorporated in PDE culture media are highly dependent on the tissue of origin (summarized in **Table 1**). When comparing the studies that used tissue specific supplements, there was no clear conclusion as to whether they improved longevity (22, 26, 31). However, Naipal, Verkaik (22) directly compared four types of media, two breast cancer-specific and two common culture media combinations, and found that the tissue specific media (listed in **Table 1**) was most effective. For HNSCC, Lee, You (26) reported that PDEs remained viable for 10 days, longer than the 2-6 days reported for other HNSCC PDE cultures in more basic media (25, 38). By contrast, in breast and pancreatic PDE studies the addition of tissue specific supplements did not improve viability compared to less complex culture media formulations (22, 31).

It is apparent that a combination of sodium bicarbonate, HEPES (4-(2-hydroxyethyl)-1-piperazineethanesulfonic acid) and cysteine has a positive effect on viability of PDE cultures across several tumor types. Gerlach, Merz (25) and Jiang, Seo (8) used these supplements in HNSCC and pancreatic cancer studies, respectively, where both had increased longevity compared to other studies. Sodium bicarbonate and HEPES are pH buffering reagents used in culture to control acidity (39). Cysteine is an essential amino acid important for protein synthesis and other metabolic functions, and helps promote proliferation in cancer cells (40). Zhang, van Weerden (41) found that a tissue-specific composition and media containing sodium bicarbonate helped improve viability compared to non-tissue-specific formulations when using PDX material. Given the fundamental roles of these supplements, they are likely to also be relevant to improving the longevity of explants for multiple tumor types, although this remains to be tested.

### Culturing Techniques

Several culturing techniques have been adopted across tumor streams for PDE culture. The three most commonly used techniques are free-floating culture, grid or pore membrane supports, and gelatin sponge supports (42). Additionally, specialized platforms such as hydrogels and co-culture systems have been investigated in improving longevity of PDE cultures (26, 43, 44).

Free-floating culture of PDEs in the absence of rotation has been largely dismissed due to the overgrowth of cells resulting in monolayer growth (42), increase in apoptosis, and loss of tissue integrity (41). However, incorporating orbital shaking into this approach reduces these issues. Ovarian PDEs were still viable, proliferating, and metabolically active at the end of a 30-day orbital shaking, free-floating culture period, and no necrotic core was present indicating successful diffusion of nutrients (17). Similarly, Naipal, Verkaik (22) adopted this technique for breast cancer PDEs, suggesting it was most effective at nutrient diffusion and easy to replicate. By contrast, Davies, Dong (28) compared free-floating under rotation to a pore membrane in breast and prostate PDX cultures finding that the membrane culture was more successful at improving viability. Histological examination, and immunohistochemistry (IHC) for E-cadherin were performed in this study, where floating cultures showed the presence of more vacuoles, loss of E-cadherin, reduced proliferative capacity, and increased apoptosis compared to the membrane cultures, confirming that membrane cultures were able to maintain structural integrity over a longer culture period (28).

The use of a grid or pore membrane has been most adopted (19, 28). The most common membrane is the Millipore system due to its open access system allowing for easy transfer and access to culture media, enabling efficient gas exchange between the PDEs and culture media (25, 27, 31). Millipore inserts coated in collagen matrix derived from rat tail have also been used to culture pancreatic PDEs (8, 15). Lim, Chang (15) commented that the precoated membrane increased adherence of the tissue to the surface. However, there was no significant increase in viability using the precoated membrane compared to standard Millipore inserts.

The use of gelatin sponges that support the PDE at the air-liquid interface is a relatively new culture technique. Centenera, Raj (42) noted that sponges help the exchange of nutrients and waste by acting as a capillary surrogate in prostate PDE cultures. Centenera, Hickey (20) and Kokkinos, Sharbeen (16) also used sponges for PDE culturing, demonstrating a significant increase in viability compared to the other culture techniques, such as filter and membrane cultures in prostate and pancreatic PDE studies, respectively. The use of sponges aids in avoiding overgrowth of a cell monolayer from cells disseminating from the explant and helps maintain homeostasis through the exchange of nutrients and waste (20).

Hydrogels that mimic features of the extracellular matrix in tissue culture have become increasingly popular, due to progression from 2D to 3D culture platforms. A preliminary study by Hribar, Wheeler (43) investigated the feasibility of embedding PDEs in VersaGel^®^, a growth factor-free hydrogel. After three days tumor cells moved out of hydrogel-embedded explants, which was associated with remodeling of the extracellular matrix and mimicking tumor invasion. An advantage of hydrogels is the ability to retain secreted growth factors and to incorporate tissue-specific growth factors into the scaffold (45). These advantages allow for a highly customizable functionalized platform for PDE growth that can be used across tumor types.

Specialized experiments have been performed requiring unique culturing techniques that have not been widely adopted due to complexity and/or technical challenges. Given that angiogenesis is a hallmark of cancer and contributes to the complexity of the TME, this is a critical component missing in routine PDE cultures. Bazou, Maimon (44) used vasculature beds formed from co-culturing endothelial and smooth muscle cells to mimic angiogenetic formation in pancreatic cancer, resulting in PDE viability extending longer than most other culture systems at 3 weeks. Similarly, Lee, You (26) positioned HNSCC PDEs on a cell sheet comprised of matched patient epithelial and sub-epithelial cells to create a co-culture system. The results showed improved cell viability and longevity of PDEs when comparing the co-culture system to other non-matrix models and showed a reduction of viable cancer cells following SOC treatments. Despite the promise of these unique culture techniques, they are not readily available, difficult to establish, and expensive, making them a less attractive technique for widespread use.

### Incubation Conditions

There is limited data addressing different cell culture incubation conditions, with most studies using 37°C, 5% CO_2_ and 21% oxygen. There is no published data addressing variation of temperature or CO_2_ concentration, however the effect of different oxygen levels on PDE viability has been examined. Misra, Moro (31) compared hyperoxic (41%), normoxic (21%) and hypoxic (less than 21%) oxygen levels concluding that hypoxic oxygen levels led to tissue degradation, but there was no significant difference in viability, proliferation, hypoxia, and metabolic activity between normoxic and hyperoxic conditions. Additionally, Davies, Dong (28) showed that lung and prostate PDX explants maintained in atmospheric oxygen (21%) had improved viability compared to tissues cultured under low oxygen conditions. Thus, atmospheric oxygen is likely to be most suitable for PDE incubation.

## Genomic Sequencing for Personalized Therapeutic Options

The successful implementation of a precision medicine pipeline into clinical practice requires a robust patient-proximal model incorporating a timely analysis of each patient’s specific actionable genomic aberrations (typically identified through WES). Using the identification of genetic aberrations to tailor personalized patient care has become increasing popular, however, it is important to understand if this is able to be incorporated into healthcare systems and be utilized as a platform for the broader community (46). The molecular screening and therapeutics (MoST) trial is currently investigating the feasibility of incorporating molecular screening to assist in informing patient management (47). Previous studies have demonstrated that the combination of WES with functional testing of actionable mutations is feasible across multiple tumor types (48, 49). Incorporating WES outputs into a precision medicine pipeline resulted in enrolment of a patient, with metastatic lung adenocarcinoma and rapid progression on SOC therapy, in a phase 1 clinical trial of a cyclin-dependent kinase 4 (CDK4) inhibitor leading to an objective response (as per response evaluation criteria in solid tumors (RECIST) criteria) (49). This demonstrated how WES can assist in finding potential treatment alternatives for advanced cancers refractory to SOC therapies. Similarly, the Zero Childhood Cancer program have utilized whole genome sequencing (WGS) in combination with cell and PDX models, further demonstrating the feasibility of this platform (50, 51). Applying PDEs to screen drugs targeting actionable mutations may present a useful preclinical drug efficacy model.

A key problem associated with the broader application of a PDE platform is misalignment of the time required for comprehensive genomic analysis and PDE longevity. The turnaround time associated with receiving WES results is highly dependent on the technology used, and thus differs across studies, but is generally 3 to 8 weeks (48, 52). This is longer than the typical viable period of PDEs cultures, which has been reported to range from 1 to 30 days dependent on tumor type (**Table 1**). Preliminary studies have investigated cryopreservation as a method to improve longevity, where Ricciardelli, Lokman (30) demonstrated that cryopreserved tissue remained viable after re-culturing. This indicates that this method could be used for long-term storage of PDEs until sequencing results are returned to the treating clinicians. PDEs have been used to test a number of chemotherapies, targeted therapies, and immunotherapies targeting molecular mechanisms identified during the WES process (**Table 2**).

**Table 2:** Comparison of drug treatments in tumor explant cultures (PDEs and PDXEs) across tumor streams.

Tumor type	Tissue source	Treatment	Treatment Period	Measure	References
Brain	PDE	temozolomide^#^	3 days	Ki67 IHC (proliferation)	(19)
				Cleaved caspase-3 IHC (apoptosis)	
				γH2AX immunofluorescent marker (DNA damage)	
				Propidium iodide immunofluorescent marker (dead cells)	
	PDE	temozolomide^#^ and irradiation^#^	3 days	Ki67 IHC (proliferation)	(18)
				Proliferation-associated gene expression	
Breast	PDXE	trastuzumab (Herceptin), docetaxel (Sigma-Aldrich)	2 days	MTT Assay (cellular metabolic activity)	(53)
				Ki67 IHC (proliferation)	
				Cleaved caspase-3 IHC (apoptosis)	
				Tumor Volume (size comparison)	
	PDE	doxorubicin^#^, tamoxifen^#^	7 days	Sirius red stain (collagen structure)	(21)
				Collagen IV IHC (collagen deposition)	
				MIB-1 IHC (proliferation)	
				Caspase-cleaved cytokeratin 18 (M30) IHC (apoptosis)	
	PDE	FAC (5-fluorouracil, adriamycin [doxorubicin], cyclophosphamide)^#^	3-5 days	TUNEL Assay (apoptosis)	(22)
				EdU uptake (proliferation)	
Colorectal	PDE	cetuximab (Merck Serono), trastuzumab (Roche), MK0752 (Selleck)	3 days	Cell counting colorimetric assay	(54)
				Ki67 IHC (proliferation)	
				Cleaved caspase-3 IHC (apoptosis)	
	PDE	oxaliplatin^#^, cetuximab^#^, pembrolizumab^#^	3 days	Ki67 IHC (proliferation)	(55)
				Cleaved caspase-3 IHC (apoptosis)	
				Hematoxylin and eosin stain (nuclei count)	
				Elastica Van Gieson stain (elasticity marker)	
	PDE	5-fluorouracil (Medac), oxaliplatin (Sanofi-Aventis)	2 days (5-fluorouracil), 22hrs (oxaliplatin) & 2hrs (5-fluorouracil)	Hematoxylin and eosin stain (nuclei count)	(56)
				Cytokeratin IHC (tumor marker)	
				Ki67 IHC (proliferation)	
Endometrial	PDE	pembrolizumab^#^, combination of carboplatin and paclitaxel^#^	1 day	Ki67 IHC/IF (proliferation)	(57)
				cPARP IHC/IF (apoptosis)	
				Cytokeratin IF (tumor marker)	
Gastrointestinal	PDE	5-fluorouracil (Medac), cisplatin (Neocorp)	2-4 days	Cytokeratin IF (tumor marker)	(58)
				Cleaved caspase-3 IF (apoptosis)	
Head and Neck Squamous Cell Carcinoma	PDXE	BYL719 (AdooQ Bioscience), erlotinib (MedChem Express), cetuximab (Merck), cisplatin (Teva Pharmaceutical Industries), olaparib (MedChem Express), 5-fluorouracil (Sigma)	1 day	Ki67 IHC (proliferation)	(38)
				TUNEL Assay (apoptosis)	
				Tumor volume	
	PDE	lupeol (Sigma-Aldrich)	3 days	Ki67 IHC (proliferation)	(59)
				Cleaved caspase-3 IHC (apoptosis)	
				p53 IHC (p53 mutation identification)	
				Cyclin D1 IHC (cell cycle)	
				CDKN2A IHC (cell cycle)	
	PDE	cisplatin (Sigma-Aldrich), docetaxel (Sanofi-Aventis Deutschland GmbH), cetuximab (Merck Serono)	3-5 days	Ki67 IHC/IF (proliferation)	(25)
				Cleaved caspase-3 IHC/IF (apoptosis)	
				Apoptotic DNA fragmentation	
				Cytokeratin IF (epithelial cell marker)	
				γH2AX IF (DNA damage)	
				Hematoxylin and eosin stain (nuclei count)	
				IBA1 IF (cell phenotype)	
	PDE	cisplatin, docetaxel (Sigma-Aldrich)	7 days	Calcein-AM and propidium iodide staining (LIVE/DEAD Assay)	(26)
				LOX-1 IF (hypoxia)	
				Green fluorescent Protein (GFP) IF (transfection efficiency)	
	PDE	cetuximab (Merck), sorafenib (Bayer)	2 days	Ki67 IHC (proliferation)	(24)
				CK5/6 IHC (cancer cell cytoplasm)	
				p40 IHC (squamous cell marker)	
				AE1/3 IHC (epithelial cell marker)	
				Collagen IHC (tumor stroma)	
Lung	PDE	cisplatin (Sigma-Aldrich), TRAIL	1 day	Laser ablation	(27)
				cPARP IHC (apoptosis)	
				Ki67 IHC (proliferation)	
				MNF116 IHC (cell phenotype)	
				p53 IHC (p53 mutation identification)	
	PDE	cisplatin (Medac GmbH), paclitaxel (Medac GmbH), nivolumab (Opdivo)	3 days	Ki67 IHC (proliferation)	(29)
				Multicolor flow cytometry (T cell response)	
				Cytokeratin IHC (tumor marker)	
				cPARP IHC (apoptosis)	
				CD3 IHC (T cell marker)	
				CD8 IHC (Cytotoxic T cell marker)	
				PD-L1 IHC (Checkpoint marker)	
				FoxP3 (Regulatory T cell marker)	
				Multispectral Imaging (T cell response)	
Ovarian	PDE	carboplatin, paclitaxel (Fresenius Kabi)	2 cycles of 24 hour treatment at day 0 and day 7 of culture	Ki67 IHC (proliferation)	(17)
				Cleaved caspase-3 IHC (apoptosis)	
				Resazurin reduction capacity assay (cell viability)	
	PDE	carboplatin (Hospira), combination of 4-MU (Sigma-Aldrich) and carboplatin (Hospira)	2 – 5 days	Ki67 IHC (proliferation)	(30)
				Cleaved caspase-3 IHC (apoptosis)	
Pancreatic	PDE	staurosporine, cycloheximide (Sigma-Aldrich)	6hrs - 1 day	Ki67 IHC (proliferation)	(8)
				Cleaved Caspase 3 IHC (apoptosis)	
	PDE	abraxane (Specialised Therapeutics)	9 days	Cytokeratin IHC (tumor marker)	(16)
				α-smooth muscle actin IHC (smooth muscle marker)	
				phospho-histone H3 IHC (proliferation)	
				BrdU uptake (proliferation)	
				CD45 IHC (lymphocyte marker)	
				Synaptophysin IHC (neuroendocrine marker)	
				TUNEL immunofluorescent marker (apoptosis)	
				Picrosirius red IHC (collagen)	
				Star 3 + Cy5-siRNA IHC/IF (therapy uptake)	
	PDE	staurosporine (Roche Diagnostics), gemcitabine (Abcam), cisplatin (Abcam)	1-2 days	Ki67 IHC (proliferation)	(15)
				Cleaved caspase-3 IHC (apoptosis)	
Prostate	PDXE	enzalutamide (Sequoia Research Products), olaparib (Selleck Chemicals)	6 days	EdU uptake (proliferation)	(41)
				TUNEL Assay (apoptosis)	
				Ki67 IHC (proliferation)	
				Androgen receptor IHC (drug target, cell phenotype)	

BrdU, Bromodeoxyuridine; EdU, 5-ethynyl-2’-deoxyuridine; IF, immunofluorescence; IHC, immunohistochemistry; PDXE, patient-derived xenograft explant; PDE, patient-derived explant, TUNEL, terminal deoxynucleotidyl transferase-mediated dUTP nick end labeling; TRAIL, TNF-related apoptosis-inducing ligand; ^#^Source of material is not indicated in publication.

### Chemotherapy

PDEs have been widely used for the evaluation of tumor response to chemotherapeutics, modeling clinical treatments that patients may receive as SOC (30, 56, 60). The possibility of resistance to chemotherapies means it is useful to test multiple regimens, where they are available, to determine the treatment(s) most likely to be effective for the individual patient. Merz, Gaunitz (19) treated glioblastoma PDEs derived from different tumors with temozolomide, a first line SOC agent, for 3 days, and found that individual tumors displayed different susceptibility to the treatment, consistent with clinical observations. Additionally, Ricciardelli, Lokman (30) treated PDEs with SOC chemotherapies such as carboplatin, which induced apoptosis in PDEs sensitive to chemotherapies but not in chemoresistant PDEs. This reinforces the relevance of adopting pipelines to probe patient-proximal models prior to clinical therapies.

### Immunotherapy

Immunotherapies have increasingly become first-line for advanced cancers such as melanoma (61), colorectal (62) and lung (63, 64), due to outstanding tumor control in a subset of patients and better tolerability when compared to chemotherapy (65). While in its infancy, a growing number of tumor types, including endometrial (57), colorectal (54, 55) and head and neck (24, 38) cancer have adopted *ex vivo* treatment of PDEs to study CTL recruitment and immunotherapy efficacy. Seo, Jiang (66) showed that pancreatic ductal adenocarcinoma cell killing within the slice was mediated by CTL activity following 6-days of combined PD-1 and C-X-C chemokine receptor type 4 (CXCR4) blockade. Interestingly, anti-PD-1 (nivolumab) treatment of PD-L1^+^ melanoma PDEs increased the inter-cell distance between T cell subsets, demonstrating mechanisms whereby CTLs may avoid Treg-mediated immunosuppression in the TME following immunotherapy (67). As the numbers of studies incorporating PDEs with immunotherapy grows, future pipelines could be assessed in parallel with multiple mRNA and protein readouts to assess immunological response in patients eligible for anti-PD-1, anti-PD-L1 or anti-CTLA-4 treatment, as described in the Lombard Street Approach (68).

### Targeted Therapy

Therapies focusing on exploiting specific molecular components to destroy proliferating cancer cells (69) are an important component of successful personalized therapeutics testing using PDEs. Although the concept of targeted therapies is more attractive than non-specific systemic treatments, side effects and resistance remain issues (70, 71). In that regard, targeted therapies are ideally informed by a WES pipeline to identify suitable and relevant candidates. Any molecular targets identified can be compared to FDA approved target therapies using *ex vivo* testing. Currently the feasibility of incorporating precision target therapies with functional testing of individual patient samples is poor due to the misalignment of WES turnaround times (48, 52, 72) and short-term viability associated with PDEs (**Table 1**). Nonetheless, targeted therapies such as erlotinib (EGFR inhibitor) and BYL719 (PI3K inhibitor) were shown to suppress the mitogen-activated protein kinase (MAPK) and protein kinase B (AKT) pathways respectively in PDEs (38). These signaling pathways are commonly upregulated during the tumorigenesis process, stimulating increased proliferation of cancer cells (73). PDE platform testing approaches can be used to determine whether such therapies might be useful for individual patients and avoid using treatments to which tumor cells are resistant and thus unnecessarily toxic.

The clinical predictive value of PDE culture as a patient-proximal testing platform needs further investigation. Brijwani, Jain (54) and Majumder, Baraneedharan (74) have used an *ex vivo* platform, based on PDE drug response, genomic and machine learning (CANscript), to predict patient responses in locally advanced/metastatic CRC and HNSCC. Their data reports that the sensitivity and specificity of this platform for clinical prediction exceeds 90% (54, 74).

## Conclusion

PDEs provide a patient-proximal model that reflect intra- and inter-tumor heterogeneity. Most tumor types have limited viability timeframes with current PDE methodologies. To improve viability across tumor types, a combination of culturing techniques and media additives can be used to maintain a physiologically relevant microenvironment *ex vivo*. Importantly, PDEs have been proven to be an effective personalized testing platform, however the feasibility of incorporating WES in this pipeline is limited until either PDE longevity is extended or cryopreservation approaches can be used to store tissues until sequencing results are available.

## Author Contributions

AT wrote the manuscript with contributions from all authors. All authors contributed to the manuscript and were involved in revisions and proof-reading. All authors approved the submitted version.

## Funding

This research was supported by funding from a Princess Alexandra Research Foundation award (EW, IV, ET, PT), and the Medical Research Future Fund (MRFF) Rapid Applied Research Translation Program (Centre for Personalised Analysis of Cancers (CPAC; EW, ET, IV: Grant ID GA59729); LB acknowledges the support of grant 1159637 awarded through the 2018 Priority-driven Collaborative Cancer Research Scheme and co-funded by Cancer Australia and Leukemia Foundation of Australia; The Translational Research Institute receives support from the Australian Government.

## Conflict of Interest

The authors declare that the research was conducted in the absence of any commercial or financial relationships that could be construed as a potential conflict of interest.

## Publisher’s Note

All claims expressed in this article are solely those of the authors and do not necessarily represent those of their affiliated organizations, or those of the publisher, the editors and the reviewers. Any product that may be evaluated in this article, or claim that may be made by its manufacturer, is not guaranteed or endorsed by the publisher.
